# Usability and Feasibility Assessment of a Social Assistive Robot for the Older People: Results from the GUARDIAN Project

**DOI:** 10.3390/bioengineering11010020

**Published:** 2023-12-24

**Authors:** Giulio Amabili, Elvira Maranesi, Arianna Margaritini, Marco Benadduci, Federico Barbarossa, Sara Casaccia, Henk Herman Nap, Roberta Bevilacqua

**Affiliations:** 1 IRCCS INRCA Scientific Direction, 60124 Ancona, Italy; g.amabili@inrca.it (G.A.); a.margaritini2@inrca.it (A.M.); m.benadduci@inrca.it (M.B.); f.barbarossa@inrca.it (F.B.); r.bevilacqua@inrca.it (R.B.); 2 Department of Industrial Engineering, Università Politecnica delle Marche, 60121 Ancona, Italy; s.casaccia@staff.univpm.it; 3 National Expertise Centre Long-Term Care, Vilans, 3527 GV Utrecht, The Netherlands; h.nap@vilans.nl

**Keywords:** social robot, older adults, ambient assisted living, ecosystem, usability, acceptance

## Abstract

In Italy, many people aged over 65 cannot live independently, causing an overall decrease in their quality of life and a need for social and health care. Due to the lack of both formal and informal caregivers, technological solutions become of paramount importance in this scenario. This article describes the user-centered development of the GUARDIAN ecosystem, consisting of a social robot integrated with two mobile applications which aim to monitor, coach, and keep the older user company in order to prolong his/her independence at home. In particular, the advancements from the alpha to the beta prototype of the ecosystem are described, achieved through the feedback collected from 41 end users—older people and their carers—that have tested the system for 6 weeks. By enhancing human–robot interaction, a positive improvement in terms of usability and acceptability of the system was retrieved. However, to increase the perceived usefulness and the impact on older users’ lives, it is necessary to make the entire system more customizable, and more capable in providing support for daily activities.

## 1. Introduction

The Italian population is among the oldest in the world, boasting a life expectancy of 82.4 years and with those aged 65 and over constituting 23.2% of the entire population [1]. Already, both the national healthcare system and the older people’s relatives are under pressure in caring for them [2]. At the same time, as their age increases it becomes more and more complicated for older people to maintain their independence to continue living in their own homes. To date, 29% of older people live alone, which is a condition that fosters fragility [3]. By considering three factors such as loneliness, the aging of the population, and the lack of caregivers, the difficulty in assisting older people (both for the healthcare system and families) is expected to worsen in the next years. One way to counteract the decline in the quality of life of older people is to coach them towards an active and healthy lifestyle. In fact, the scientific literature provides plenty of evidence that coaching, leading a healthy life, regularly taking prescribed medication, constantly monitoring the elderly person’s condition, and maintaining a daily routine all have a positive impact on the elderly person’s quality of life and aging [4]. However, these activities require the work and commitment of various people, from family members to healthcare professionals, and end up taking a toll on their lives. Therefore, it is desirable to transfer at least part of the mentioned activities to automated systems, which are able to support the daily life of the elderly and at the same time relieve the stress of their caregivers.

### 1.1. Background

For this purpose, there is strong evidence that technology should be an integral part of the solution for older people’s quality of life [5,6], through assistive devices that enable older people to maintain independence for as long as possible, and at the same time, optimize the work of caregivers, whether formal or informal. Today, the use of these technologies with the elderly is growing rapidly and examples can be found for a wide variety of purposes: to treat people with severe dementia [7,8], to counteract cognitive impairment [9,10], and to maintain a healthy lifestyle for active aging [11,12].

However, a recurring problem with the adoption of technology in the older population is the acceptability and usability of explored solutions [13,14]. Recent studies in the field have shown that the use of social assistive robots (SARs) may increase engagement with these technologies [15,16,17]. Given the peculiarity of SARs, i.e., providing assistance and helping to achieve progress in several domains through the establishment of a close and effective interaction with the user [18], they are suitable for coaching older people. However, current research highlights how the usability and acceptability of technology, including SARs, are still challenges in elderly care [19]. Taking this into account, along with the need for technological solutions to address the above issues, designing and assessing new systems is necessary for facing upcoming challenges in the field of elderly assistance.

### 1.2. The GUARDIAN Project

The aim of this paper is to present the Italian results of the European GUARDIAN project (AAL-2019-6-120-CP), focused on the development of an ecosystem based on the social robot Misty II (by Misty Robotics, mistyrobotics.com/misty-ii) and two mobile applications, implemented over three waves of iterative design [20]. The project aimed to build an ecosystem capable of supporting both older people and caregivers in three European countries: Italy, Switzerland, and the Netherlands. The main goal is to prolong the older people’s independence in their own home by acting as a companion that can take over tasks from caregivers. Moreover, GUARDIAN aimed to support both formal and informal caregivers, by allowing them to monitor the older person remotely and by creating a care network around the older user: in this way, the care network could communicate with the user and share information among each other. The design and development of the ecosystem is described by Ciuffreda et al. [21].

### 1.3. Aim of the Paper

This paper focuses on how the technical development carried out from prototype 2 to prototype 3 brought effects on the usability and acceptability of older users. The aim is to evaluate the acceptability, the usability, and the impact of the GUARDIAN system on older Italian users and how these aspects have changed in the light of the transition from prototype 2 to prototype 3.

### 1.4. Structure of the Paper

The continuation of the paper is structured as follows: in Section 2, prototype 3 development is described in detail along with a general overview of the GUARDIAN ecosystem; then, the study design and the related approval by ethical committee are reported in Section 2.2 and Section 2.7, respectively. In addition, in Section 2, participants’ involvement, scales and questionnaires asked, and statistical analysis are detailed (Section 2.3–2.6). In Section 3, both quantitative and qualitative results are shown with the help of tables and figures. The findings are finally discussed in Section 4, along with limitations of the study and future works.

## 2. Materials and Methods

### 2.1. GUARDIAN Prototype 3

**Figure 1:**
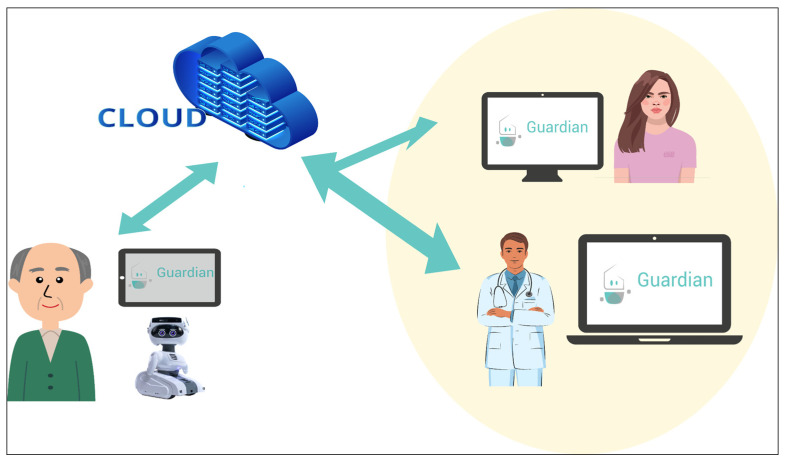
GUARDIAN system overview.

The GUARDIAN ecosystem, aimed to prolong older people’s independence in their homes, is composed of 3 main technologies: the social robot Misty II and two mobile applications: the Senior App (SA) intended for the older user and the Caregiver App (CA) dedicated to the user’s care network (i.e., relatives and healthcare professionals who take care of the older user). The role of GUARDIAN is to monitor the older user, send him/her reminders, provide suggestions, and keep him/her in touch with caregivers. In fact, through the ecosystem a bidirectional communication between the older user and the caregivers is established. In particular, a bidirectional communication is established for the senior thanks to the combination of Misty and the Senior App, which is intended to coach, motivate, and remind him/her of certain actions. Misty also acts as a social companion in order to engage the user. The caregiver app, instead, is designed to allow caregivers to set reminders, to monitor, and to send messages to the loved one. The overall framework of the GUARDIAN ecosystem is pictured in Figure 1.

The core of GUARDIAN is the social robot Misty, which is able to interact with older people. User localization is integrated into the system architecture, combining passive and active methods. Indeed, Misty is able to localize the user in indoor environments thanks to its onboard sensors, particularly its three omnidirectional microphones. These microphones capture audio data, enabling the extraction of the Direction of Arrival to pinpoint the user’s location. Eye contact capability is also implemented, enabling Misty to engage with users and express emotions. Once the user is located—by periodic activation of microphones—Misty looks at him/her, and if the case, it speaks aloud a reminder or a suggestion.

The SA is a mobile app connected with the social robot, establishing one-way communication between Misty and older adults. In detail, the reminder or suggestion appears on the tablet screen along with possible answers, if the case.

The third and last component is the Caregiver Application (CA) that creates a two-way channel between seniors and caregivers to enable remote interaction. In this way, caregivers can monitor well-being, offer reminders, and receive feedback, while seniors can respond through Misty or a visible component in the senior application. Reminders are displayed on the SA with response options, and the feedback is collected and viewed by caregivers in the CA. The set of caregivers associated with a user form the user’s care network, in compliance with the GUARDIAN user-centric approach.

The GUARDIAN system was developed by following the ISO 13047—User Centered Design [20]. According to this standard, 3 prototypes were released during the project, so that new functionalities and services could be added after any test, based on users’ feedback. Prototype 1 (P1) was released and evaluated with a usability in lab test, as presented by Ciuffreda et al. (2023) [19]. Then, prototype 2 (P2) and 3 (P3) were respectively assessed after 6 weeks with a first study—alpha test—and a second one—beta test. On the basis of the feedback collected on P2, the existing functionalities were improved, and new ones were added in P3. Therefore, the qualitative data collected during the alpha test were translated into technical requirements for P3, leading to the development of the features listed in Table 1 and presented in detail in the following subsections. The shift from P2 to P3 aimed to make GUARDIAN smarter, and it concerned four major functionalities: a smarter reminder system, the introduction of sleep mode and a snooze function, and the improvement of eye-based and gesture-based communication.

#### 2.1.1. Smart Reminder System

To meet the need for postponing reminders in case of no answer or user request, it was essential to consider other incoming reminders and their timing, as reminders might overlap or queue up within a short time frame. To address these issues, a robust algorithm has been developed. Additionally, for those reminders that are time-sensitive (e.g., medication and meal reminders), P3 included a mechanism to avoid infinite snoozing. To support this, the reminder database structure has been updated to include three new fields:

- Reminder delay: specifies how long to delay the next reminder.
- Maximum snoozes: determines the maximum number of times a user can snooze a reminder (with the option to set it to 0 to disable snoozing).
- Past reminder time: tracks how many times the reminder has already been displayed.

Initially, these fields are populated with default values by the Caregiver Application backend and are subsequently monitored and updated by the Cloud. Moreover, a queue system has been implemented in the Cloud to handle scenarios where multiple reminders are scheduled for the same time. In such cases, only the oldest reminder is presented, with the others scheduled to appear immediately after the first one has been successfully sent or has expired.

On the Senior Application the snooze button appears on each reminder page, if set. If the user did not react to the reminder within three minutes, and if the snooze for that reminder is set, then the reminder is automatically sent again. In this way, the reminder flow is optimized.

#### 2.1.2. Eye Contact Skill

The eye contact skill enables the robot to autonomously establish eye contact with a person when they are present and track their movements. This ability is pivotal in creating a sense of being observed, which is an important initial step in fostering interaction. Moreover, the eye contact allows users to discern when the robot is attentive to them. This aspect was reinforced by feedback collected during alpha tests. In P2, when the robot no longer detects a person’s face, it will first glance around, in case the person moves out of its line of sight. In case the robot still cannot detect a face, it transitions into standby mode.

Due to the relevance and high appreciation of eye contact skill reported by users, a further development and improvement was planned. In fact, even though the primary function of the eye contact skill remained largely consistent with P2, significant efforts were dedicated to enhancing its integration with the Senior App and Cloud services in P3:

- the intensity of the “look around” behavior was reduced in response to feedback indicating that it was perceived as excessive and made some individuals feel overly scrutinized. In P3, the robot now performs the “look around” action only once before returning to standby mode more promptly.
- the implementation of a sleep mode, allowing the robot to become unresponsive and refrain from providing messages. This feature is user-controlled, as opposed to autonomous control: when the robot is in sleep mode or engaged with the senior app, the eye contact skill is deactivated. In idle periods, the eye contact skill is activated, and the robot can operate in standby, normal, or alert modes. The new standby state excludes the “look around” behavior, but portrays a slightly sleepy expression, in contrast to being completely asleep when the system-wide sleep mode is activated. In Table 2 the different states of the eye contact skill are reported along with the associated functionalities:

Also, the robot’s eye expressions and movements were increased and improved in P3 development. This feature utilizes animated eye images supplied by the robot’s manufacturer and relies on motorized arms integrated within the robot. The specific expressions and movements employed in this feature are detailed in Table 3. This capability is implemented using the JavaScript language, and communication with the robot is facilitated through Misty’s HTTP API, which governs the robot through HTTP requests.

#### 2.1.3. Sleep Mode

A new feature was necessary to enhance the acceptance of the system by older users, who were concerned about the increasing cost of energy due to the Ukraine war in the first half of 2022. The power-saving need was also in line with the responsible innovation focus of the GUARDIAN project. For this reason, the sleep mode feature was introduced to grant users the capability to temporarily turn off the entire system. This new feature also aimed to relieve users of privacy concerns. However, the system could not remain offline indefinitely. For these reasons, a solution was devised to accommodate both needs: seniors could put the system to sleep at their discretion, and the system would automatically awaken at predetermined times, according to users’ preferences. Additionally, the system could enter sleep mode at a predetermined time if wished. In this sleep mode, the system ceases recording any data, including the presence of the senior, while still sending reminders. To implement this, extensive modifications were made to various aspects of the system.

In fact, a new status was introduced in the Message Queuing Telemetry Transport (MQTT) channel, shared among the Cloud, the robot, and the Senior App. This status preserves the actual wake-up time of the system for each client. This was accomplished by utilizing the retain functionality of MQTT, storing the last value for future reference. The Cloud updates the system’s components when there is a request to transition to sleep or wake up. A schedule was established to wake up the systems at specific times. The wake-up action involves altering the status and issuing commands to the robot to change its posture.

The system was adapted to check the “weakness” status of the system and prevent the sending of reminders when the system is in sleep mode. The Cloud is capable of reading the retained status on the MQTT broker and responding accordingly.

When a senior triggers the capacitive sensors to activate sleep mode for the robot, the robot responds by lowering its head and closing its eyes, as shown in Table 3. Specifically, the head movement is controlled using an API for the yaw angle of the robot’s head, while the closing of the robot’s eyes is managed through the screen and animated eye images provided by the robot’s manufacturer.

A new user interface (UI) was developed for the asleep Senior Application. In this mode, the tablet displays a predominantly black screen with an image of sleeping Misty eyes and the usual connectivity status indicators. This UI was designed to be unobtrusive, as the black background significantly reduces brightness and saves energy. A “wake-up” button is provided to transition back to the awake status. When the senior presses the button, the new status is relayed to the Cloud for the appropriate system response, and the Senior Application returns to the standard UI. The Senior Application consistently monitors this status and switches to the “asleep” UI as soon as it detects the transition to sleep mode. It remains vigilant in sleep mode to promptly switch back to the normal UI if a change is initiated from other sources.

### 2.2. Study Design

The study protocol was published by Maragaritini et al. [18]. The alpha and beta pilot tests consisted of testing, respectively, the P2 and the P3 of the GUARDIAN platform with 5 and 10 dyads, composed of an older adult and an informal caregiver. Both tests lasted for 6 weeks: the alpha test was run between March and May 2022, while the beta pilot test was run from September to December 2022. In particular, due to the availability of only 5 Misty robots, 5 out of 10 participants tested the system from September to October, whereas the remaining 5 users interacted with GUARDIAN in November and December.

### 2.3. Inclusion and Exclusion Criteria

The participants were recruited according the protocol, assessing whether or not they matched inclusion and exclusion criteria, listed as follows:

1. Older adults:
  - Aged over 65 years old;
  - No previous diagnosis of mild cognitive impairment or dementia;
  - Cognitive integrity (Mini Mental State Examination ≥ 24);
  - Have an informal caregiver;
  - Healthy sight and hearing;
  - Good written and oral comprehension of the local language.

2. Informal caregivers:
  - Relatives or close friends of a senior;
  - Providing frequent support/care on a daily or weekly basis;
  - Aged more than 18 years old.

3. Formal caregivers:
  - Home care nurses, general practitioners, or health professionals involved in the daily/weekly care of a frail senior;
  - At least 1 year of work experience.

### 2.4. Participants’ Involvement

The participants were contacted through the IRCCS INRCA hospital in Ancona, in collaboration with the rehabilitation unit, and by consulting a list of people who participated in previous projects. The participants were fully informed about the project, about the phase of testing. All participants signed the informed consent before starting the testing.

### 2.5. Scales and Questionnaires

The aim of both alpha and beta testing was to assess the usability and the acceptability of prototypes 2 and 3 of the GUARDIAN platform, respectively. In order to do that, ad hoc questionnaires and qualitative interviews on usability, acceptability, and usefulness were asked at the end of the testing. The usability ad hoc questionnaire is the primary outcome of this study and consists of 19 statements about ease of use, acceptability, and usability of the system. The user-friendliness ad hoc questionnaire is focused on the robot, consisting of 11 statements about ease of use, likeability, and satisfaction about Misty. The ad hoc questionnaire on impact is about how the system helped the older user in the supported ADL. It consists of 10 statements. Every aforementioned questionnaire was scored by assigning a 7-point Likert scale, where 7 means strongly agree with the item and 1 means strongly disagree with the item.

Due to the low number of users, qualitative analysis was also conducted, by asking about the most and least appreciated features of the system, how the system impacted the users’ daily life, the perceived usefulness, and suggestions for further improvements.

### 2.6. Statistical Analysis

Quantitative data have been analyzed through RStudio 2023.09.0 Build 463 and Microsoft Excel 2016. In view of the limited amount of data available and the type of data, i.e., discrete scales (ad hoc questionnaire), the normality of the distribution was first analyzed by means of the Shapiro–Wilk test and then the following tests were conducted: if the normality hypothesis was verified, the parametric Student’s *t*-test [22] was applied; otherwise, the non-parametric Mann–Whitney U-test [23] was applied. The aforementioned tests were applied to compare alpha and beta tests results (P2 vs. P3), but also the results between the alpha and the second part of the beta test (P2 vs. P3 without bugs), since important technical issues in P3 were solved during the experimentation. These issues caused frequent disconnections of the system, seriously affecting the user experience.

### 2.7. Ethical Approval

The presented study strictly followed the Declaration of Helsinki guidelines [24]; indeed, it was approved by the IRCCS INRCA Ethical Committee on 21 October 2021 and then published on clinicaltrials.gov with the identifier NCT05284292 [25].

## 3. Results

### 3.1. Participants

Throughout the two experimental phases of the GUARDIAN project, a total of 41 people participated in the study. The whole sample constituted 15 older adults, 15 informal caregivers, and 11 formal caregivers. The alpha test was run with 11 participants (5 older adults, their informal caregivers, and 1 formal caregiver), whereas the beta test was run with 30 participants equally distributed among the three end user categories. However, only one formal caregiver tested the system for 4 months following all the users. The sample was gender balanced, and as expected, the educational level and the technological competence was much higher among (in)formal caregivers than older adults. Details about participation and demographic data are presented in Table 4.

For the alpha pilot test, five older adults were recruited, of whom only one was male. They were aged 74.4 (±8.9) years, with an average MMSE score = 27.6 (±2.3), and four of them were used to receiving informal care regularly.

A similar population was recruited for the beta pilot test, where the cohort comprised ten older adults, evenly split between five females and five males, with an average age of 75.4 (±5.8) years and an average MMSE score = 29.9 (±0.3). One of them dropped the test after the first week because he did not consider the platform congenial to his needs. None of the seniors received formal professional care, but they regularly consulted doctors, either neurologists or family physicians, at least once a month. Additionally, nine seniors received frequent informal care, primarily from their children, with seven of them receiving assistance from their sons on a regular basis. Notably, three of these seniors had their sons visit them daily, primarily aiding in transportation and providing companionship to them. The average technological competence of the older adults was low as expected: between 3 and 4 on a 5-point Likert scale.

The group of informal caregivers consisted of five females and five males, with an average age of 47.9 (±10.5) years. These informal caregivers rated their technological competence, on average, as 4.6 (±0.7) on a 5-point Likert scale. On the other hand, there were ten formal caregivers, equally divided between five females and five males, with an average age of 39.3 (±13) years. The formal caregivers, like their informal counterparts, also rated their technological competence as 4.6 (±0.5) on a 5-point Likert scale.

### 3.2. Usability Results

The goal of the beta test was to assess the usability of the GUARDIAN platform after 6 weeks of use through an ad hoc usability questionnaire, shown in Table 5. The items that received the best scores (i.e., the highest scores) are about the comfortability of the system and the organization and the availability of the information. The users were generally satisfied, and appreciated how easy the system was to learn and to use. Also, the interface, which included Misty, was highly rated by the participants. On the other hand, none of the older users appreciated the error message functionality for fixing problems. Looking deeply at the table below, it can be seen as the informative content and the appearance of the system improved with prototype 3, whereas users’ expectations were not met. Moreover, the ease of use was still high. However, no statistically significant difference, neither for any item nor for the global score, was found between the two test results (*p* > 0.05).

As mentioned in Section 2.2, the prototype 3 was tested in two different time windows. In between the two time windows, bugs in P3 were fixed. For this reason, half of the participants of beta test experienced the system with bugs, while the second half benefitted of bug fixing. In fact, the introduction of new skills from P2 to P3 led to an increase in system disconnections, experienced by five older users (from IT_01 to IT_05). Due to bug fixing, five older users (from IT_06 to IT_10) benefited from the updated version. The first five older users who tested P3 in the first months struggled with disconnection problems, so their feedback was highly affected by this malfunctioning. For this reason, in Table 6 the comparison between P2 and P3 after bug fixing is reported. As almost every item improved after the update, and this result is coherent with the expectations: the enhancement of HRI in P3, together with the resolution of bugs that emerged as a result of development, has improved the usability of GUARDIAN. However, no statistical difference was found for any item, but it can be due to the very low number of the sample (five users for P2 against four users for P3).

### 3.3. Robot User Friendliness

Similar findings resulted from the ad hoc questionnaire to assess the user friendliness of the robot which was asked at the end of the test (T2). In fact, as shown in Table 7, the best scores (i.e., highest scores) were given to questions related to the ease of use and the interface. On the contrary, the usefulness and the impact of the robot on daily life were poorly rated. However, there is a significant difference between the two versions: *p* < 0.05.

### 3.4. Impact on Daily Life

The difference due to bug fixing was also remarked upon in the ad hoc questionnaire on the impact of GUARDIAN. In fact, participants who benefitted from the last updated version of P3 found the system much more helpful and impactful than those who struggled with disconnection issues, as shown in Table 8.

Despite the bug fixing, no significant improvement has been noticed between P3 and P2, as shown in Table 9. Older users were significantly more satisfied and confident with P3 than P2. However, according to the older users, the impact of GUARDIAN on independence, activity level, and daily activities did not increase. This result is in line with the expectations, according to which enhancing HRI positively impacts on general usability, acceptability, and so satisfaction about a system, but it is not enough to make the system impactful for the users.

### 3.5. Qualitative Results

Participants were asked to describe their experience during the beta test of P3. Four of the five users who tested P3 before the bug fixing lamented frequent disconnections and crashes of the system that made GUARDIAN useless in their opinion. The remaining five older users affirmed to have followed all the suggestions and the reminders that Misty delivered throughout the 6 weeks. However, one of them pointed out that he needed to be close to Misty when a reminder has been delivered, otherwise he was not able to listen to it. All the users did not find GUARDIAN effective for social connectedness, but some of them suggested to implement a video call functionality to easily contact their loved ones.

Answering the question “What do you appreciate the most about GUARDIAN?”, three old users mentioned the ease of use, three stated that Misty is nice and/or funny, two said that the robot kept company with them, and one user found the participation in the project stimulating to learn new things. When the participants were asked to list the difficulties encountered throughout the testing, eight out of nine pointed out disconnection issues. In particular, the five older users who tested P3 before bug fixing stressed that point: “From a technical point of view it was not working well. Maybe it’s me who doesn’t know how to use it, but it should be made for a person like me, not for a technological genius, otherwise what’s the point of offering it to the elderly?” said one of them. One of the older users who interacted with GUARDIAN P3 before bug fixing was able to understand when to restart both the tablet and the robot to make it work again; however, she was disappointed by the number of times that this operation was needed. Finally, three older users stated a major concern as that due to disconnections and frequent technical failures, the medication reminders were not sent. In their opinion, if GUARDIAN failed in that goal, then the system is completely useless.

When participants were asked if the system changed their daily pattern, all the older users answered negatively. Then, when asked if, in their opinion, GUARDIAN will become important for the existing home care system, most of them were skeptical. However, some of them gave inputs on how to make GUARDIAN more impactful: “it should be supplemented with something that could, for example, detect physiological parameters, or keep the person in contact with an emergency system” one older user said. Another user stated: “tt should be expanded, and the tablet should be removed”. The other three users wished for improvements, but they did not go into detail on which functions to add.

**Figure 2:**
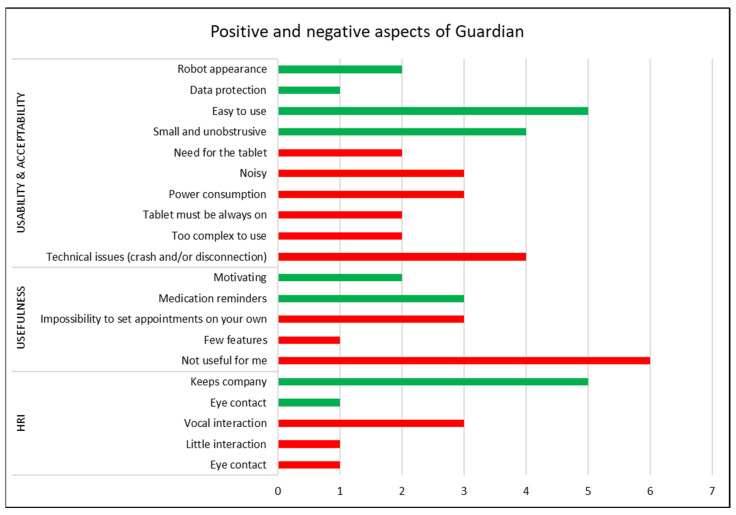
Positive (green) and negative (red) aspects of GUARDIAN prototype 3 mentioned by older users.

Finally, participants were asked to list three positive and three negative aspects about GUARDIAN. The results are shown in Figure 2, where green and red bars correspond to positive and negative aspects respectively. The length of the bar is proportional to the number of users who mentioned the aspect (x-axis). The aspects are also grouped by area of interest (left column): usability, usefulness, and human–robot interaction. This qualitative analysis is coherent with results presented in this and the above sections, where the appearance of Misty and the ease of use are the most appreciated aspects among the users. Moreover, five of them perceived Misty as a companion. The most appreciated function was the medication reminder. However, six out of nine participants did not find GUARDIAN useful for them. About usability, disconnection issues were the most negative aspect, but users were also concerned about power consumption, despite the improvements made from P2 to P3. Probably, their concern came from the noise from Misty’s fan when in charge. In fact, three users mentioned the noise as a negative aspect. This analysis also confirmed the desire of interacting vocally with Misty, avoiding the use of the tablet (Senior App).

## 4. Discussion

Prototype 3 of the GUARDIAN ecosystem was well received in terms of acceptability and usability by elderly users in Italy. In line with expectations and the study hypothesis, this study demonstrates that the co-design of technological systems based on iterative end user involvement and the introduction of a social robot both work to promote final user acceptance. In fact, the processing of the feedback received during the testing of prototype 2 and the subsequent declination into technical requirements to be developed in order to improve the ecosystem, made the users appreciate the whole system more. The aspects on which the greatest differences emerged are those where action was taken between the two tests (alpha and beta): snooze function, smart reminder system, eye contact, and sleep mode. The sleep mode in particular allows us to highlight how following the iterative user-centered co-design method is effective in taking into account and solving problems that are difficult to foresee, but no less relevant from the user’s point of view. A specific case in point is the sudden increase in energy prices (caused by the sudden Ukrainian war), which has particularly sensitized the elderly population in Italy. Precisely in the light of this need that emerged during the alpha tests, it was decided to develop a sleep mode that would allow significant energy savings, and which overall brings sustainability and responsible innovation to the entire GUARDIAN ecosystem, as it applies both for Misty and the Senior App. Furthermore, given the high degree of user appreciation for Misty, due to her affable and friendly behavior which was particularly manifested through her eye contact skills, it was decided to further improve this aspect of HRI with the introduction of new expressions and gestures. Lastly, as the feature deemed most useful by P2 users had been the reminder feature, which at the same time had detected critical issues in operation, it was decided to significantly improve the sending of reminders, making the whole process more intelligent. Through the introduction of the snooze function, but also the improvement of user detection, by means of the integration of photo analysis to the existing audio analysis, the delivery of reminders proved to be more efficient and therefore liked by users.

Despite the technical advances made to the GUARDIAN ecosystem in its latest prototype, unresolved problems remain. Chief among them is the perceived usefulness. Although Misty appears to be welcome and easy to use, the system seems to be ineffective in significantly improving users’ daily lives. This problem could have two explanations, both of which are plausible. First, healthy and independent patients were recruited in the trial-following the inclusion and exclusion criteria—who do not feel the need for external intervention in their lives at the time of the trial. Second, a limitation is that the functions that can be performed by the system (sending reminders, delivering messages, suggesting certain activities, asking about daily activities) appear to be insufficient and too repetitive to significantly impact users’ lives. According to the end users’ wishes of a more personalized gadget and their self-perception as healthy people, the coaching function should be enhanced. For instance, GUARDIAN could provide recommendations about physical exercise, nutrition, and social life according to end users’ needs and habits. One piece of very recent research shows a high interest by older people for this kind of services and their wish for personalization [26]. On the other side, this upgrade would mean a re-design of the whole system, starting from personal data needed to train and run a new machine learning algorithm.

### 4.1. Lessons Learnt

The present study confirmed that enhancing HRI is beneficial for usability and acceptability, but it is not sufficient to increase the perceived usefulness. The adoption of social and/or humanoid robots is confirmed to be potentially successful for building technological ecosystems for older adults’ assistance at home. Moreover, developing advanced skills for making the robot’s behavior as human-like as possible, is highly appreciated. Finally, the integration of two different applications—one for the older user and the other for the care network—to create a bidirectional communication flow is efficient. However, the older population’s expectations are increasing in terms of technology, as they wish to communicate freely with the robot and want increasingly personalized solutions. Providing personalized reminders and scheduled suggestions may not be enough to fully engage and coach older people, to prolong their independence at home.

### 4.2. Limitations

The presented study aimed to focus on how the technical development of a social robot-based solution impacts on usability and acceptability in older people. However, due to the poor number of participants (9) and the limited time of experimentation (6 weeks), the results cannot be generalized. Furthermore, the quantitative results are based on ad hoc questionnaires. Finally, the statistical analysis performed on this data was affected by the low number of participants. No deeper analysis could be conducted for this reason.

### 4.3. Future Work

In future, a new research study should explore the positive indications arising from GUARDIAN, such as the performance and the development of Misty, and the connected Caregiver App. In addition to that, free vocal interaction, made possible by recent large language models, and more ADL-related content should be implemented to make ecosystems like GUARDIAN very impactful for older people’s independence. As the user-centered iterative co-design approach resulted in efficient shifting from P2 to P3, feedback collected in this study should be highly considered for innovative solutions in the assistance of older people at home.

On a more conceptual perspective, GUARDIAN and similar systems should be much more impactful in order to jump from research to real world applications, due also to the lack of high quality studies [27]. In fact, as regards practical managerial significance, i.e., the inclusion of a wider demographic, the system should meet multiple needs. To date, many technological systems have been designed for specific population (e.g., affected by one pathological condition), and offer a quite narrow spectrum of functionalities [28,29]. As emerged from the present study, end users ask for personalized and heterogeneous functionalities embedded in a single ecosystem, so that they can use it according to their needs. On the basis of this analysis, it would be desirable in the future to start from the great acceptability shown by the elderly population towards Misty II, to build an upgraded ecosystem which should be rich in functionality, so as to create a scalable product capable of meeting the individual needs of many elderly people struggling to maintain their independence.

## Figures and Tables

**Table 1:** Technical developments implemented for P3.

Services	Technical Development
Take photo *	Integrated to audio-based location better locate the user.
Sleep mode	Prevent reminders and interaction when system is in sleep mode;Dark screen on the senior app.
Snooze function *	Added the possibility to snooze the reminder; Repeat reminder after half an hour no response is given.
Send message to the user *	The robot spoke aloud the messages sent.
Sense touch *	Touching the robot head it is possible to switch from sleep to active mode and viceversa.
Eye contact *	Robot moves and changes eye images as the user answers.
Variance in gestures and facial expression	More eye images and arm movements added.
Sleep mode *	The system is in power saving mode and it is not responsive
Follow up question	Added more answer options (also in case of positive answers)

The star (*) indicates that the service was new to P3. Other services were already integrated in P2, but some improvements were provided by adding to the skills in the right column.

**Table 2:** Eye contact skill states and associated functionalities.

State	Listen to MQQT Cloud Commands	Time to Revert to Lower State	Number of Times to Look Around	Facial Expression	Color of Chest Led	Face Detection
OFF/Standby	Yes	-	-	-	Off	-
ON/Normal Alert	Yes	20 s	2		White	Yes
	Yes	10 s	3		Green	Yes

**Table 3:** Eye images and movements implemented in P3 to increase the human–robot interaction in monitoring and reminder services.

Monitoring and Reminders Action	Functions	Developed Movement	Animated Eye Images
Well-being	When the robot understands: “yes”	Move the head up and down and then return to the neutral position (3 s).	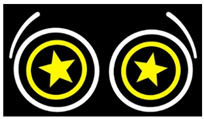
Medication	When the robot asks for medication report		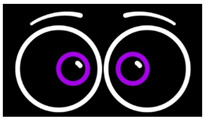
	When the robot understands that the senior has taken the medications	Move the head up and down and then return to the neutral position (3 s).	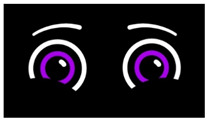
Meal	When the robot asks for meals report	Both hands in the air for 4 s. After the default condition.	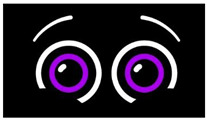
Sleep	When the robot asks for a sleep report	Left arm up and after 3 s neutral position	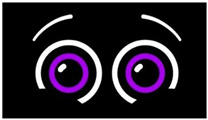
		Arms up and down and after 3 s neutral position	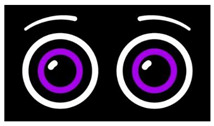
Activity suggestions	When the robot suggests activities	Both hand in the air and after 3 s neutral position	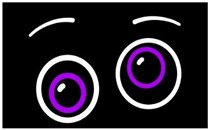
Extra features	When the robot activates the localization	Look right, center, left and after 3 s neutral head position	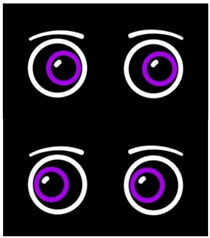
Sleep mode	Simulate a sleep mode Move the head down	Simulate a sleep mode Move the head down	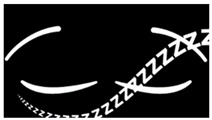

**Table 4:** Demographic data of participants.

Phase	Data	Older Adults	Informal Caregivers	Formal Caregivers
Alpha	Participants, n	5	5	1
	Age (years), mean (SD)	74.4 (8.9)	39.6 (13.7)	30
	MMSE, mean (SD)	27.6 (2.3)	-	-
	Female, n (%)	4 (80%)	3 (60%)	0 (0%)
	Male, n (%)	1 (20%)	2 (40%)	1 (100%)
	Living alone, n (%)	2 (40%)	-	-
	Educational level, mean (SD)	3.6 (1.1)	4.8 (0.4)	5.0
	Technological competence, mean (SD)	3.6 (1.3)	4.6 (0.5)	5.0
Beta	Participants	10	10	10
	Age (years), mean (SD)	75.4 (5.8)	47.9 (10.5)	39.3 (13.0)
	MMSE, mean (SD)	29.9 (0.3)	-	-
	Female, n	5 (50%)	5 (50%)	5 (50%)
	Male, n	5 (50%)	5 (50%)	5 (50%)
	Living alone, n (%)	3 (30%)	-	-
	Educational level, mean (SD)	3.2 (1.3)	4.3 (0.7)	4.8 (0.4)
	Technological competence, mean (SD)	3.3 (1.7)	4.6 (0.7)	4.6 (0.5)
Total	Participants, n	15	15	11
	Female, n (%)	9 (60%)	8 (53.3%)	5 (45.5%)
	Male, n (%)	6 (40%)	7 (46.7%)	6 (54.5%)

N = number, SD = standard deviation. The educational level has been computed according to the following scale: 1 = no education; 2 = primary school; 3 = middle school; 4 = high school; 5 = degree or above. The technological competence has been assessed according to the following Likert 5-points scale: 1 = no competence; 2 = very little; 3 = little; 4 = some; 5 = a lot; MMSE = Mini-Mental State Examination.

**Table 5:** Usability ad hoc questionnaire scores of P2 and P3 (alpha and beta tests respectively).

Items	P2	P3	
	Mean (SD)	Mean (SD)	Diff
Overall, I am satisfied with how easy it is to use.	5.40 (2.61)	5.11 (1.27)	−0.29
It was simple to use.	5.20 (2.49)	5.11 (1.27)	−0.09
I could (effectively) successfully complete the tasks and scenarios using the system.	5.60 (2.61)	4.56 (1.88)	−1.04
I was able to complete the tasks and scenarios quickly using the system.	5.20 (1.92)	4.78 (1.79)	−1.42
I was able to efficiently complete the tasks and scenarios using the system.	5.20 (1.92)	4.78 (1.79)	−1.42
I feel comfortable using the system.	4.80 (2.68)	6.11 (0.78)	+1.31
It was easy to learn to use.	4.80 (2.68)	5.44 (1.01)	+0.64
I believe I could become productive quickly using the system.	5.40 (1.34)	4.00 (1.32)	−1.40
The system gave error messages that clearly told me how to fix problems.	4.60 (3.29)	1.56 (0.73)	−3.04
Whenever I made a mistake, I could recover easily and quickly *	4.60 (2.30)	3.56 (1.42)	−1.04
The information (such as online help, on-screen messages) provided was clear.	3.40 (3.29)	4.44 (1.81)	+1.04
It was easy to find the information I needed.	4.20 (2.17)	5.56 (0.88)	+1.36
The information provided was easy to understand.	4.50 (2.65)	6.11 (0.78)	+1.61
The information was effective in helping me complete the tasks and scenarios.	4.20 (2.28)	5.33 (0.87)	+1.13
The organization of information on screens was clear.	4.60 (3.29)	6.33 (0.50)	+1.73
The interface was pleasant.	4.00 (2.55)	6.56 (0.73)	+2.56
I liked using the interface of the system. *	3.60 (1.95)	5.33 (1.50)	+1.73
Overall, I am satisfied with the system.	4.60 (1.34)	3.89 (1.60)	−0.71
This system has all the functions and capabilities I expect it to have.	4.60 (2.88)	2.44 (0.88)	−2.16

Scale from 1 to 7, where 1 stands for “strongly disagree with the item” and 7 stands for “strongly agree with the item”; SD = standard deviation. The star (*) indicates when the item scores were normally distributed for both groups.

**Table 6:** Comparison between usability ad hoc questionnaire scores for P2 and P3 after bug fixing.

Items	P2	P3 Bugs Fixed	
	Mean (SD)	Mean (SD)	Diff
Overall, I am satisfied with how easy it is to use.	5.40 (2.61)	5.75 (0.50)	0.35
It was simple to use.	5.20 (2.49)	5.75 (0.50)	0.55
I could (effectively) successfully complete the tasks and scenarios using the system.	5.60 (2.61)	5.75 (0.50)	0.15
I was able to complete the tasks and scenarios quickly using the system.	5.20 (1.92)	6.00 (0.00)	0.80
I was able to efficiently complete the tasks and scenarios using the system.	5.20 (1.92)	6.00 (0.00)	0.80
I feel comfortable using the system.	4.80 (2.68)	6.00 (0.00)	1.20
It was easy to learn to use.	4.80 (2.68)	5.75 (0.50)	0.95
I believe I could become productive quickly using the system.	5.40 (1.34)	5.00 (0.82)	−0.40
The system gave error messages that clearly told me how to fix problems.	4.60 (3.29)	2.25 (0.50)	−2.32
Whenever I made a mistake, I could recover easily and quickly. *	4.60 (2.30)	4.50 (1.00)	−0.10
The information (such as online help, on-screen messages) provided was clear.	3.40 (3.29)	5.75 (0.96)	2.35
It was easy to find the information I needed.	4.20 (2.17)	5.75 (1.26)	1.55
The information provided was easy to understand.	4.50 (2.65)	6.00 (0.82)	1.50
The information was effective in helping me complete the tasks and scenarios.	4.20 (2.28)	6.00 (0.82)	1.80
The organization of information on screens was clear. *	4.60 (3.29)	6.50 (0.58)	1.90
The interface was pleasant. *	4.00 (2.55)	6.50 (0.58)	2.50
I liked using the interface of the system. *	3.60 (1.95)	5.50 (1.00)	1.90
Overall, I am satisfied with the system.	4.60 (1.34)	4.75 (1.50)	0.15
This system has all the functions and capabilities I expect it to have.	4.60 (2.88)	3.00 (0.82)	−1.60

Scale from 1 to 7, where 1 stands for “strongly disagree with the item” and 7 stands for “strongly agree with the item”; SD = standard deviation. The star (*) indicates when the item scores were normally distributed for both groups.

**Table 7:** User friendliness ad hoc questionnaire variation for P3: comparison between the results with and without disconnection issue versions at T2.

Statement	P3 with Bugs	P3 Bugs Fixed	Difference	P3
	Mean (SD)	Mean (SD)		Mean (SD)
It was easy to use the robot.	3.40 (2.61)	6.50 (0.58)	3.10	4.78 (2.49)
I feel comfortable while using the robot.	3.40 (2.61)	6.25 (0.50)	2.85	4.67 (2.40)
It was easy to learn it how to use the robot.	4.20 (1.92)	6.00 (0.82)	1.80	5.00 (1.73)
The given information about the robot was easy to understand.	5.20 (2.05)	7.00 (0.00)	1.80	6.00 (1.73)
The interface of the robot was pleasant.	6.20 (1.10)	6.75 (0.50)	0.55	6.44 (0.88)
I liked to use the robot.	3.00 (2.35)	5.50 (0.58)	2.50	4.11 (2.15)
The robot has all the features and capabilities I expected.	2.80 (1.30)	4.75 (0.96)	1.95	3.67 (1.50)
The robot has an influence on me.	1.80 (1.79)	3.50 (0.58)	1.70	2.56 (1.59)
The robot is important to me personally.	1.80 (1.79)	3.50 (1.00)	1.70	2.56 (1.67)
The robot makes me reconsider certain habits such as my diet, exercise pattern or medication intake.	1.6 (1.34)	3.00 (1.41)	1.40	2.22 (1.48)
Overall, I am satisfied with the robot.	2.2 (2.17)	5.00 (0.82)	2.80	3.44 (2.19)

Scale from 1 to 7, where 1 stands for “strongly disagree with the item” and 7 stands for “strongly agree with the item”; SD = standard deviation.

**Table 8:** P3 results for the ad hoc questionnaire on impact.

Statement	P3 with Bugs	P3 Bugs Fixed	Difference	Overall
	Mean (SD)	Mean (SD)		Mean (SD)
Using GUARDIAN helps me take my medication on time.	1.8 (1.3)	3.5 (1.0)	1.7	2.44 (1.51)
Using GUARDIAN encourages me to be more active.	1.2 (0.4)	2.0 (0.8)	0.8	1.56 (0.73)
Using GUARDIAN helps me eat and/or drink enough.	1.2 (0.4)	2.0 (1.4)	0.8	1.56 (1.01)
Using GUARDIAN makes me less lonely.	1.8 (1.8)	2.0 (1.2)	0.2	1.89 (1.45)
Using GUARDIAN makes me more independent.	1.2 (0.4)	2.0 (0.8)	0.8	1.56 (0.73)
GUARDIAN helps me have a daily routine.	1.4 (0.9)	2.3 (1.3)	0.9	1.78 (1.09)
GUARDIAN helps me inform my caregivers about my well-being.	1.6 (0.9)	2.5 (1.3)	0.9	2.00 (1.12)
Using GUARDIAN makes me feel safer.	1.4 (0.9)	2.3 (1.0)	0.9	1.78 (0.97)
I feel confident while using GUARDIAN.	3.2 (0.4)	3.8 (0.5)	0.6	3.44 (0.53)
Overall, I am satisfied with GUARDIAN.	1.8 (1.3)	3.5 (1.0)	1.7	3.00 (1.73)

Scale from 1 to 5, where 1 stands for “strongly disagree with the item” and 5 stands for “strongly agree with the item”; SD = standard deviation.

**Table 9:** Comparison between P2 and P3 without bugs for ad hoc questionnaire on impact.

Statement	P2	P3 without Bugs	Difference
	Mean (SD)	Mean (SD)	
Using GUARDIAN helps me take my medication on time.	3.60 (1.67)	3.50 (1.00)	−0.10
Using GUARDIAN encourages me to be more active.	2.40 (1.34)	2.00 (0.82)	−0.40
Using GUARDIAN helps me eat and/or drink enough.	2.00 (1.41)	2.00 (1.41)	0
Using GUARDIAN makes me less lonely.	3.40 (2.19)	2.00 (1.15)	−1.40
Using GUARDIAN makes me more independent.	2.00 (1.73)	2.00 (0.82)	0
GUARDIAN helps me have a daily routine.	2.80 (1.79)	2.25 (1.26)	−0.55
GUARDIAN helps me inform my caregivers about my well-being.	3.40 (3.40)	2.50 (1.29)	−1.10
Using GUARDIAN makes me feel safer.	-	2.25 (0.96)	-
I feel confident while using GUARDIAN.	-	3.75 (0.50)	-
Overall, I am satisfied with GUARDIAN.	-	3.50 (1.00)	-

Scale from 1 to 5, where 1 stands for “strongly disagree with the item” and 5 stands for “strongly agree with the item”; SD = standard deviation.

## Data Availability

The data collected during the usability tests are owned by the GUARDIAN consortium partners. The data are not publicly available because the data contains information that could compromise research participants’ privacy. Data is however available from the authors upon reasonable request and permission from the GUARDIAN project coordinator, Henk Herman Nap.

## References

[1] ISTAT. https://www.istat.it/demografiadelleuropa/bloc-1c.html?lang=it.

[2] Gagliardi C., Piccinini F., Lamura G., Casanova G., Fabbietti P., Socci M. (2022). The Burden of Caring for Dependent Older People and the Resultant Risk of Depression in Family Primary Caregivers in Italy. Sustainability.

[3] ISTAT. https://www.istat.it/it/archivio/14562.

[4] Bevilacqua R., Casaccia S., Cortellessa G., Astell A., Lattanzio F., Corsonello A., D’Ascoli P., Paolini S., Di Rosa M., Rossi L. (2020). Coaching Through Technology: A Systematic Review into Efficacy and Effectiveness for the Ageing Population. Int. J. Environ. Res. Public Health.

[5] McKee K., Matlabi H., Parker S.G. (2012). Older People’s Quality of Life and Role of Home-Based Technology. Health Promot. Perspect..

[6] Stara V., Soraci L., Takano E., Kondo I., Möller J., Maranesi E., Luzi R., Riccardi G.R., Browne R., Dacunha S. (2023). Intrinsic Capacity and Active and Healthy Aging Domains Supported by Personalized Digital Coaching: Survey Study among Geriatricians in Europe and Japan on eHealth Opportunities for Older Adults. J. Med. Internet Res..

[7] Bevilacqua R., Maranesi E., Felici E., Margaritini A., Amabili G., Barbarossa F., Bonfigli A.R., Pelliccioni G., Paciaroni L. (2023). Social robotics to support older people with dementia: A study protocol with Paro seal robot in an Italian Alzheimer’s day center. Front. Public Health.

[8] Lu L.C., Lan S.H., Hsieh Y.P., Lin L.Y., Lan S.J., Chen J.C. (2021). Effectiveness of Companion Robot Care for Dementia: A Systematic Review and Meta-Analysis. Innov. Aging.

[9] Bevilacqua R., Felici E., Cucchieri G., Amabili G., Margaritini A., Franceschetti C., Barboni I., Paolini S., Civerchia P., Raccichini A. (2023). Results of the Italian RESILIEN-T Pilot Study: A Mobile Health Tool to Support Older People with Mild Cognitive Impairment. J. Clin. Med..

[10] Manca M., Paternò F., Santoro C., Zedda E., Braschi C., Franco R., Sale A. (2021). The impact of serious games with humanoid robots on mild cognitive impairment older adults. Int. J. Hum. Comput. Stud..

[11] Rampioni M., Moșoi A.A., Rossi L., Moraru S.-A., Rosenberg D., Stara V. (2021). A Qualitative Study toward Technologies for Active and Healthy Aging: A Thematic Analysis of Perspectives among Primary, Secondary, and Tertiary End Users. Int. J. Environ. Res. Public Health.

[12] Fattah S.M.M., Sung N.-M., Ahn I.-Y., Ryu M., Yun J. (2017). Building IoT Services for Aging in Place Using Standard-Based IoT Platforms and Heterogeneous IoT Products. Sensors.

[13] Bevilacqua R., Felici E., Cavallo F., Amabili G., Maranesi E. (2021). Designing Acceptable Robots for Assisting Older Adults: A Pilot Study on the Willingness to Interact. Int. J. Environ. Res. Public Health.

[14] Cavallo F., Esposito R., Limosani R., Manzi A., Bevilacqua R., Felici E., Di Nuovo A., Cangelosi A., Lattanzio F., Dario P. (2018). Robotic Services Acceptance in Smart Environments with Older Adults: User Satisfaction and Acceptability Study. J. Med. Internet Res..

[15] Amabili G., Cucchieri G., Margaritini A., Benadduci M., Barbarossa F., Luzi R., Riccardi G.R., Pelliccioni G., Maranesi E., Bevilacqua R. (2022). Social Robotics and Dementia: Results from the eWare Project in Supporting Older People and Their Informal Caregivers. Int. J. Environ. Res. Public Health.

[16] McDonald D.D., Walsh S., Vergara C., Gifford T., Weiner D.K. (2012). The effect of a Spanish virtual pain coach for older adults: A pilot study. Pain. Med..

[17] Gaudiello I., Zibetti E., Lefort S., Chetouani M., Ivaldi S. (2016). Trust as Indicator of Robot Functional and Social Acceptance. An Experimental Study on User Conformation to Icub Answers. Comput. Human Behav..

[18] Feil-Seifer D., Mataric M. Defining socially assistive robotics. Proceedings of the 9th International Conference on Rehabilitation Robotics.

[19] Abdi J., Al-Hindawi A., Ng T., Vizcaychipi M.P. (2018). Scoping review on the use of socially assistive robot technology in elderly care. BMJ Open.

[20] Ciuffreda I., Amabili G., Casaccia S., Benadduci M., Margaritini A., Maranesi E., Marconi F., De Masi A., Alberts J., de Koning J. (2023). Design and Development of a Technological Platform Based on a Sensorized Social Robot for Supporting Older Adults and Caregivers: GUARDIAN Ecosystem. Int. J. Soc. Robot..

[21] Naveira A.V., Masi A.D., Wacb K., Amabili G., Vastenburg M., Alberts J., Lovis C., Mantas J., Gallos P., Zoulias E., Hasman A., Househ M.S., Diomidous M., Liaskos J., Charalampidou M. (2022). Designing a Social Robot Companion to Support Homecare: Usability Results. Advances in Informatics, Management and Technology in Healthcare.

[22] Kim T.K. (2015). T test as a parametric statistic. Korean J. Anesthesiol..

[23] Mann H.B., Whitney D.R. (1947). On a test of whether one of two random variables is stochastically larger than the other. Ann. Math. Stat..

[24] World Medical Association (2013). World Medical Association Declaration of Helsinki: Ethical principles for medical research involving human subjects. JAMA.

[25] Margaritini A., Benadduci M., Amabili G., Bonfigli A.R., Luzi R., Wac K., Nap H.H., Maranesi E., Bevilacqua R. (2022). The social robot companion to support homecare nurses: The GUARDIAN study protocol. Contemp. Clin. Trials Commun..

[26] Bedaf S., Marti P., De Witte L. (2019). What are the preferred characteristics of a service robot for the elderly? A multi-country focus group study with older adults and caregivers. Assist. Technol..

[27] Boumans R., van Meulen F., Hindriks K., Mark Neerincx M., Olde Rikkert M. (2019). Robot for health data acquisition among older adults: A pilot randomised controlled cross-over trial. BMJ Qual. Saf..

[28] Pu L., Moyle W., Jones C., Todorovic M. (2019). The Effectiveness of Social Robots for Older Adults: A Systematic Review and Meta-Analysis of Randomized Controlled Studies. Gerontologist.

[29] Lee M., Tran D.T., Lee J.H. (2021). 3D Facial Pain Expression for a Care Training Assistant Robot in an Elderly Care Education Environment. Front. Robot. AI.

